# Sevoflurane but not propofol enhances ovarian cancer cell biology through regulating cellular metabolic and signaling mechanisms

**DOI:** 10.1007/s10565-022-09766-6

**Published:** 2022-10-08

**Authors:** Cong Hu, Bincheng Wang, Zhigang Liu, Qiling Chen, Masashi Ishikawa, Han Lin, Qingquan Lian, Jun Li, Jia V. Li, Daqing Ma, The ESA-IC Onco-Anaesthesiology Research Group

**Affiliations:** 1 Zhejiang Province Key Lab of Anesthesiology, Department of Anesthesiology and Perioperative Medicine, The Second Affiliated Hospital and Yuying Children’s Hospital of Wenzhou Medical University, Wenzhou, 325027 Zhejiang China; 2 Division of Anaesthetics, Pain Medicine and Intensive Care, Department of Surgery and Cancer, Faculty of Medicine, Imperial College London, Chelsea & Westminster Hospital, London, SW10 9NH UK; 3 Department of Metabolism, Digestion and Reproduction, Faculty of Medicine, Imperial College London, London, SW7 2AZ UK

**Keywords:** Inhalational anesthetic, Intravenous anesthetic, Ovarian cancer, Metabolism, HIF-1α

## Abstract

Perioperative risk factors, including the choice of anesthetics, may influence ovarian cancer recurrence after surgery. Inhalational anesthetic sevoflurane and intravenous agent propofol might affect cancer cell metabolism and signaling, which, in turn, may influence the malignancy of ovarian cancer cells. The different effects between sevoflurane and propofol on ovarian cancer cell biology and underlying mechanisms were studied. Cultured ovarian cancer cells were exposed to 2.5% sevoflurane, 4 μg/mL propofol, or sham condition as the control for 2 h followed by 24-h recovery. Glucose transporter 1 (GLUT1), mitochondrial pyruvate carrier 1 (MPC1), glutamate dehydrogenase 1 (GLUD1), pigment epithelium-derived factor (PEDF), p-Erk1/2, and hypoxia-inducible factor 1-alpha (HIF-1α) expressions were determined with immunostaining and/or Western blot. Cultured media were collected for ^1^H-NMR spectroscopy-based metabolomics analysis. Principal component analysis (PCA) and orthogonal projections to latent structures discriminant analysis (OPLS-DA) were used to analyze metabolomics data. Sevoflurane increased the GLUT1, MPC1, GLUD1, p-Erk1/2, and HIF-1α expressions but decreased the PEDF expression relative to the controls. In contrast to sevoflurane, propofol decreased GLUT1, MPC1, GLUD1, p-Erk1/2, and HIF-1α but increased PEDF expression. Sevoflurane increased metabolite isopropanol and decreased glucose and glutamine energy substrates in the media, but the opposite changes were found after propofol treatment. Our data indicated that, unlike the pro-tumor property of sevoflurane, propofol negatively modulated PEDF/Erk/HIF-1α cellular signaling pathway and inhibited ovarian cancer metabolic efficiency and survival, and hence decreased malignancy. The translational value of this work warrants further study.

**Graphical abstract:**

• Sevoflurane promoted but propofol inhibited ovarian cancer cell biology.

• Sevoflurane upregulated but propofol downregulated the GLUT1, MPC1, and GLUD1 expressions of ovarian cancer cells.

• Sevoflurane enhanced but propofol inhibited ovarian cancer cellular glucose.

metabolism and glutaminolysis.

• Sevoflurane downregulated PEDF but upregulated the Erk pathway and HIF-1α, while propofol had the adverse effects on ovarian cancer cells.

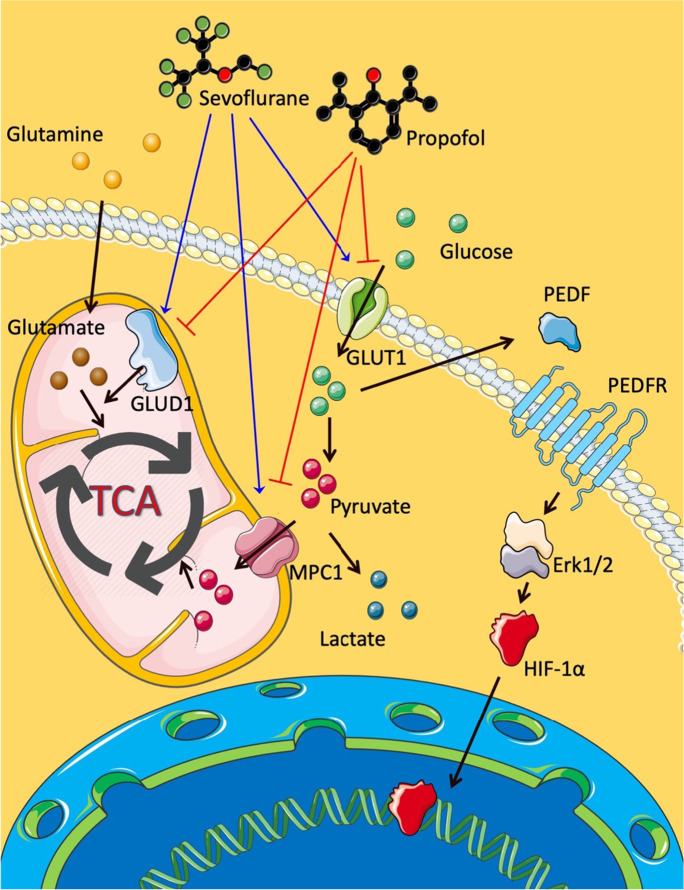

**Supplementary Information:**

The online version contains supplementary material available at 10.1007/s10565-022-09766-6.

## Background

Ovarian cancer is the eighth most diagnosed and the eighth-highest lethal cancer in women globally. There were an estimated more than 313,000 new cases and more than 207,000 deaths of ovarian cancer worldwide in 2020 (Ferlay et al. 2020). Surgery remains the frontline therapy for ovarian cancer patients, as the tumor resection at the early stage can be very effective (Wyld et al. 2015). However, ovarian cancer recurrence following surgery is still a leading cause of death (Saphner et al. 1996; Zhu et al. 2011).

Many risk factors affect cancer recurrence following surgery, and one of them may be the choice of anesthetics and anesthesia techniques (Horowitz et al. 2015). A previous publication from our laboratory demonstrated that sevoflurane increased the migration of ovarian cancer cells via upregulating *VEGFA*, *MMP11*, *CXCR2*, and *TGF-β* gene expressions (Iwasaki et al. 2016). In contrast, propofol was reported to have an “anti-tumor” property; for example, our previous study demonstrated that propofol inhibited the proliferation and migration of prostate cancer cells (Huang et al. 2014). Another study from our lab found that propofol might inhibit the biology of lung cancer cells through glucose metabolism and HIF-1α relevant pathway (Hu et al. 2021). Clinically, a retrospective study reported that patients, who had surgery for breast, colonic or rectal cancer, were under propofol-based total intravenous anesthesia or sevoflurane inhalational anesthesia. Their overall 1-year and 5-year survival rates were higher with propofol anesthesia (Enlund et al. 2014). However, whether the two different anesthetics affect cancer progression through an identical mechanism remained unknown.

Glucose is taken up by cancer cells through GLUT1 and transformed to pyruvate, which is converted to lactate rather than enters mitochondria via MPC1 to be utilized by the TCA cycle (Zou et al. 2019; Pezzuto et al. 2020). Glutamine can be transformed into glutamate and further converted to α-ketoglutarate under the catalyzation of GLUD1 for use in the TCA cycle generating crucial intermediates for cancer growth and survival (Craze et al. 2019). This process is named glutaminolysis, and GLUD1 is the crucial enzyme that locates in the inner membrane of mitochondria. Any factors that affect the GLUD1 expression and activity may interfere with cancer cell survival (Son et al. 2013).

The transcriptional factor HIF-1α plays a crucial role in the development of many tumor types, including breast, colonic, and lung cancer (Zhao et al. 2014) while the cellular signaling Erk1/2 pathway can regulate HIF-1α (Karagiota et al. 2019). Several regulators can regulate the signaling pathway of Erk1/2, and one of these regulators is the PEDF, a member of the serine protease inhibitor family. The therapeutic values of PEDF were found for choroidal neovascularization, heart disease, and cancer (Filleur et al. 2009). Besides the upstream signaling of HIF-1α, the downstream effectors of HIF-1α might also be involved in the ovarian cancer malignancy, namely, C-X-C motif chemokine ligand 12 (CXCL12) and C-X-C motif chemokine receptor 4 (CXCR4) (Takano et al. 2014; Scala et al. 2020).

Propofol and/or sevoflurane are commonly used for anesthesia for cancer surgery. We recently found that commonly used intravenous anesthetic propofol affect lung cancer cells through glucose metabolism alteration (Hu et al. 2021). It is still unknown whether commonly used inhalational anesthetic, e.g., sevoflurane, shares a similar or opposite feature relative to propofol. Investigating potential malignant properties and underlying mechanisms of different anesthetics are, therefore, urgently needed. In the current study, we compared the malignant potential of sevoflurane and propofol on ovarian cancer cells through metabolism and molecular/cellular signaling changes.

## Methods

### Cell culture

Ovarian cancer cell line (SKOV3, RRID: CVCL_0532) was purchased from ECACC (Wiltshire, UK). SKOV3 cells were cultured in Gibco RPMI media 1640 (ThermoFisher, Paisley, UK) supplemented with 10% fetal bovine serum (FBS) and 1% penicillin–streptomycin (ThermoFisher, Paisley, UK) at 37 °C with 5% CO_2_ and balanced with air. SKOV3 cells were administered with clinically relevant anesthetic concentrations of 2.5% sevoflurane (ABOTT, Sittingbourne, UK) and 4 μg/mL propofol (Sigma-Aldrich, Dorset, UK), or media (naïve control), or intralipid (vehicle control) (Santa Cruz Biotechnology, Dallas, Texas, USA) for 2 h and followed by 24-h recovery in fresh media. For the sevoflurane group, SKOV3 cells were cultured in a specially designed chamber that was filled with 2.5% sevoflurane in air balanced with 5% CO_2_ for 2 h. For propofol group, propofol was diluted with intralipid and culture media to a final concentration as 4 μg/mL exposure to culture SKOV3 cells for 2 h in air balanced with 5% CO_2_. The dose or concentration and length of exposure of these anesthetics are all clinically relevant.

### CCK-8 assay

After recovery, SKOV3 cells were incubated with media plus cell counting kit-8 (CCK-8) solution at 37 °C for 4 h. The OD values of cancer cells were detected at a wavelength of 450 nm. CCK-8 solution with culture media but without cancer cells was used as blank. Cell viability values equaled to [OD (treatment) − OD (blank)]/[OD (control) − OD (blank)] × 100%.

### Ki-67 staining

After recovery, SKOV3 cells were washed with phosphate-buffered saline (PBS) (Santa Cruz Biotechnology, Dallas, Texas, USA) and incubated in 4% paraformaldehyde (Santa Cruz Biotechnology, Dallas, Texas, USA) for 15 min. Cancer cells were washed with PBS plus 0.02% Triton X-100 (Sigma-Aldrich, Dorset, UK) 3 times for 5 min each and blocked with 10% donkey serum (Sigma-Aldrich, Dorset, UK) for 30 min. After blocking, cells were incubated with anti-Ki-67 antibody (1:200, rabbit polyclonal) (Santa Cruz Biotechnology, Dallas, Texas, USA) at 4 °C overnight and anti-rabbit antibody (1:1000, goat polyclonal) (Abcam, Cambridge, UK) for 1 h at room temperature under dark in sequence. The 4’,6-diamidino-2-phenylindole (DAPI) mounting media (Vector Laboratories, Burlingame, California, USA) were mounted on cancer cells. The fluorescent images were taken under a fluorescence microscope, and the percentage of Ki-67-positive cells was calculated by Fiji (ImageJ 2.0) software (National Institutes of Health, Bethesda, Maryland, USA).

### Wound healing assay

SKOV3 cells were seeded in petri dishes to form a continuous monolayer. Cancer cells were incubated in pure media, 2.5% sevoflurane, or 4 μg/mL propofol for 2 h. The monolayer of cancer cells was scratched to form a cell-free gap. The petri dishes were washed with PBS to discard suspended cancer cells. The cell-free gap was captured under a microscope as the baseline. After incubated with culture media for 24 h, the cell-free gap was captured again under the microscope to calculate the migration capability of cancer cells with Fiji (ImageJ 2.0) software (National Institutes of Health, Bethesda, Maryland, USA). The calculation formula was as follows: wound closure (%) = [gap area (baseline) − gap area (24 h)]/gap area (baseline) × 100%.

### Transwell assay

SKOV3 cells were seeded in petri dishes and administered with media, 2.5% sevoflurane, or 4 μg/mL propofol for 2 h. After administration, cancer cells were mixed with FBS-free media and seeded in the upper chamber of the Transwell assay kit, which was pre-embedded with Corning Matrigel matrix (Corning, New York, USA). The FBS-enriched media was placed into the lower chamber of the Transwell assay kit. After being incubated at 37 °C for 24 h, the upper chamber was inserted in the 70% methanol (ThermoFisher, Paisley, UK) for 30 min. The cancer cells on the upper chamber were dyed with 0.1% crystal violet (Sigma-Aldrich, Dorset, UK) for 15 min and washed to get away the leftover dye. The cells on the upper membrane of the upper chamber were removed. The cells on the bottom membrane of it were regarded as invasive cells and detected by a microscope. The number of invasive cells was counted with Fiji (ImageJ 2.0) software (National Institutes of Health, Bethesda, Maryland, USA).

### Immunofluorescent staining

After recovery, SKOV3 cells were washed with phosphate-buffered saline (PBS) (Santa Cruz Biotechnology, Dallas, Texas, USA) and incubated in 4% paraformaldehyde (Santa Cruz Biotechnology, Dallas, Texas, USA) for 15 min. Cells were washed with PBS plus 0.02% Triton X-100 (Sigma-Aldrich, Dorset, UK) 3 times for 5 min each and blocked with 10% donkey serum (Sigma-Aldrich, Dorset, UK) for 30 min. After blocking, cells were incubated with primary antibody at 4 °C overnight and secondary antibody for 1 h at room temperature under dark in sequence (Table S1). The DAPI mounting media (Vector Laboratories, Burlingame, California, USA) were mounted on cancer cells. The expressions of proteins were detected by a fluorescence microscope.

### Western blot analysis

After recovery, SKOV3 cells were adapted with cell lysis buffer (Cell signaling Technology, London, UK) to extract proteins. The protein samples were mixed with NuPAGE LDS sample buffer (ThermoFisher, Paisley, UK) and boiled at 95 °C for 10 min. The boiled samples were loaded onto a NuPAGE Bis–Tris gel (ThermoFisher, Paisley, UK) for electrophoresis. After electrophoresis, the protein on the gel was transferred to a nitrocellulose membrane (ThermoFisher, Paisley, UK). The membrane was blocked with 5% non-fat milk for 1 h and incubated with primary antibody at 4 °C overnight and secondary antibody on the next day (Table S1) for 1 h and adapted with luminol reagent solution A and B (Santa Cruz Biotechnology, Dallas, Texas, USA). The protein bands were detected by GeneSnap Version 7.1 (Syngene, Cambridge, UK), and the levels of protein were analyzed by Fiji (ImageJ 2.0) software (National Institutes of Health, Bethesda, Maryland, USA).

### Proton NMR spectroscopy

After recovery, the culture media of SKOV3 cells were collected. One day before the NMR experiment, media samples were thawed at room temperature. The sample with 540 μL was collected and mixed with 60 μL potassium phosphate buffer (pH = 7.4) containing 1.5 M KH_2_PO_4_, 1 mM NaN_3_, 0.1% TSP, and D_2_O. A total of 580 μL mixture was placed into an NMR tube (Bruker Corporation, Rheinstetten, Germany) with an outer diameter of 5 mm. ^1^H-NMR spectra of media samples were obtained using a Bruker 600 MHz spectrometer (Bruker Corporation, Rheinstetten, Germany) at the operating ^1^H frequency of 600.13 MHz at a temperature of 300 K. A standard NMR pulse sequence (recycle delay-90°-t_1_-90°-t_m_-90° acquisition) was applied to acquire ^1^H-NMR spectral data (t_1_ = 3 μs, t_m_ = 100 ms). The water peak suppression was achieved using selective irradiation during a recycle delay of 4 s and t_m_. A 90° pulse was adjusted to ~ 10 μs. A total of 32 scans for cell media were collected into 64 k data points with a spectral width of 20 ppm. ^1^H-NMR spectral data were acquired using TopSpin 4.0.9 (Bruker Corporation, Rheinstetten, Germany). The spectral data were imported into MATLAB R2018a (MathWorks, Natick, Massachusetts, USA) and SIMCA (Sartorius, Gottingen, Germany) for multivariate statistical analysis. The chemical shift ranged from 4.7 to 5.0, and from − 1 to 0.3 was cut. The spectral data were aligned with the recursive segment-wise peak alignment method and normalized with the probabilistic quotient normalization method (Dieterle et al. 2006; Veselkov et al. 2009). Metabolites were identified using Chenomx software (CHENOMX, Edmonton, Canada).

### Statistical analysis

Except for data of ^1^H-NMR spectroscopy analysis, the other data were presented as dot plot and mean ± standard deviation, which was analyzed by one-way analysis of variance (ANOVA) followed by the Dunnett test for comparison wherever appropriate (GraphPad Prism 8.2.0, GraphPad Software, La Jolla, California, USA). A two-sided *p* value of less than 0.05 was considered to be a statistical significance. The NMR data were imported and processed by MATLAB R2018a (MathWorks, Cambridge, UK) programming language with the in house developed MATLAB scripts (Trygg et al. 2007; Haggart 2019). After data was normalized and aligned, PCA and OPLS-DA was processed with MATLAB as well (Dieterle et al. 2006; Veselkov et al. 2009).

## Results

### Effects of sevoflurane or propofol on cancer malignancy

**Fig. 1:**
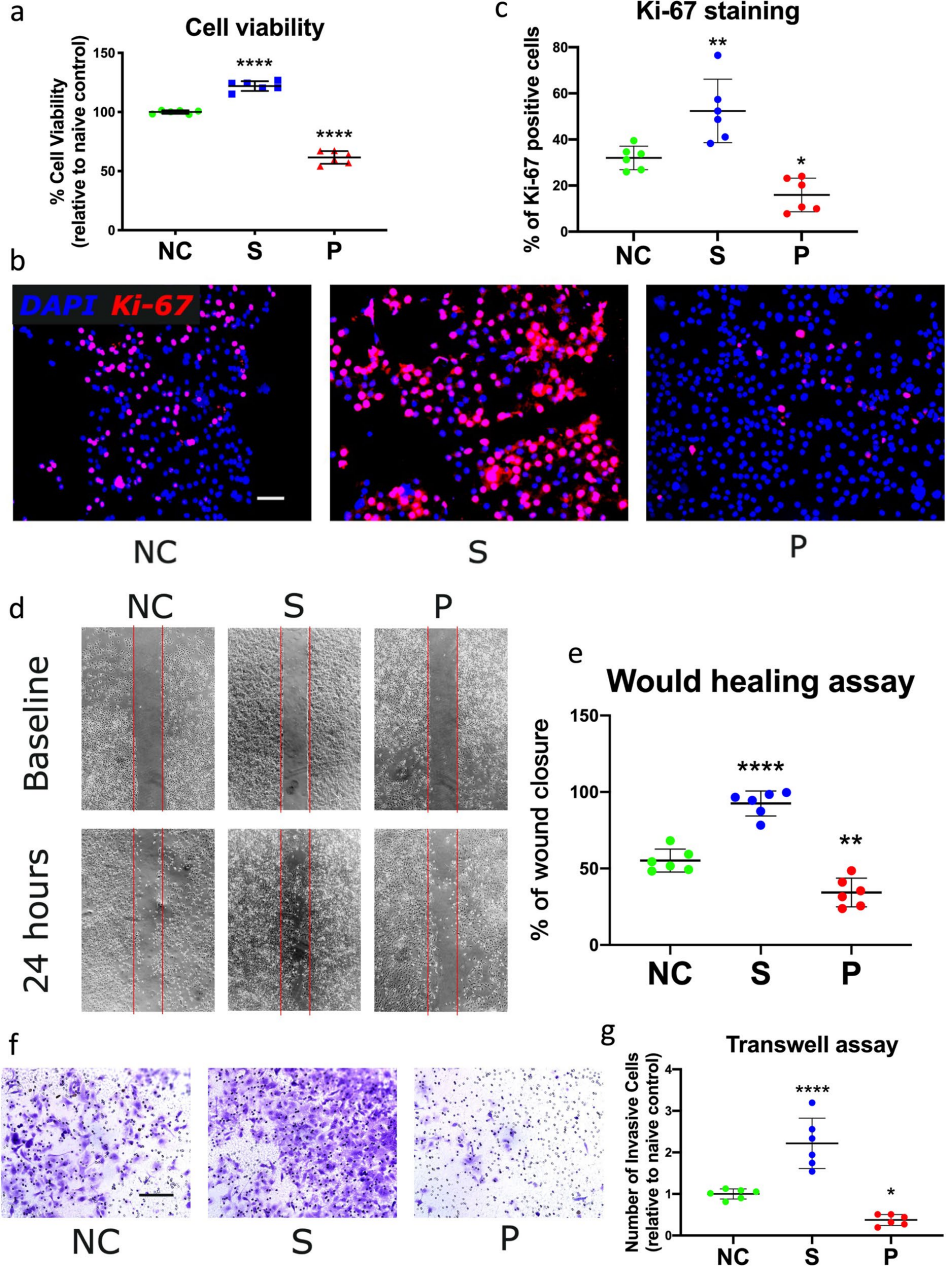
Cell viability, proliferation, migration, and invasion of ovarian cancer cells after sevoflurane or propofol administration. Ovarian cancer (SKOV3) cells were administered with pure culture media (naïve control), 2.5% sevoflurane, or 4 μg/mL propofol. The cell viability of ovarian cancer cells was evaluated with the cell counting kit-8 assay (**a**). The cell proliferation of ovarian cancer cells was evaluated by identifying Ki-67-positive cells (**b** and **c**). The migration of SKOV3 cells was evaluated by calculating gap closure with the wound healing assay (**d** and **e**). The invasion was evaluated by identifying invasive cells with Transwell assay (**f** and **g**). The data were expressed as mean ± standard deviation and dots plot (*n* = 6). *p < 0.05, **p < 0.01, ****p < 0.0001 versus naïve control. Scale bar: 100 μm. NC, naïve control group; S, sevoflurane group; P, propofol group

Sevoflurane significantly increased the ovarian cancer cell viability of compared to naïve control (NC vs. S, 100.0 ± 1.5 vs. 122.0 ± 4.1, *p* < 0.0001, *n* = 6), while propofol significantly decreased the cell viability (NC vs. S, 100.0 ± 1.5 vs. 61.6 ± 5.4, *p* < 0.0001, *n* = 6) (Fig. 1a). The proliferation of SKOV3 cells was indicated by the number of Ki-67-positive cells (Fig. 1b). It was found that the number of Ki-67-positive cells was significantly increased by sevoflurane administration (NC vs. S, 32.0 ± 5.1 vs. 52.4 ± 13.8, *p* = 0.004, *n* = 6). However, Ki-67-positive cells were significantly decreased by propofol treatment (NC vs. P, 32.0 ± 5.1 vs. 16.0 ± 7.3, *p* = 0.019, *n* = 6) (Fig. 1c).

The migrating capability of ovarian cancer cells after sevoflurane treatment was significantly greater than that of the naïve control (NC vs. S, 55.2 ± 7.5 vs. 92.5 ± 8.2, *p* < 0.0001, *n* = 6) (Fig. 1d). In contrast, the migrate capability of cancer cells was inhibited by propofol exposure (NC vs. P, 55.2 ± 7.5 vs. 34.3 ± 9.4, *p* = 0.001, *n* = 6) (Fig. 1e). The invasion of ovarian cancer cells was evaluated by comparing the number of invasive cells with the Transwell assay. Significantly higher number of invasive cells was observed in the sevoflurane group compared to naïve control (NC vs. S, 1.0 ± 0.1 vs. 2.2 ± 0.6, *p* < 0.0001, *n* = 6) (Fig. 1f), whereas propofol exhibited an opposite effect (NC vs. P, 1.0 ± 0.1 vs. 0.4 ± 0.1, *p* = 0.018, *n* = 6) (Fig. 1g).

### Effects of sevoflurane or propofol on the expressions of GLUT1, MPC1, and GLUD1

**Fig. 2:**
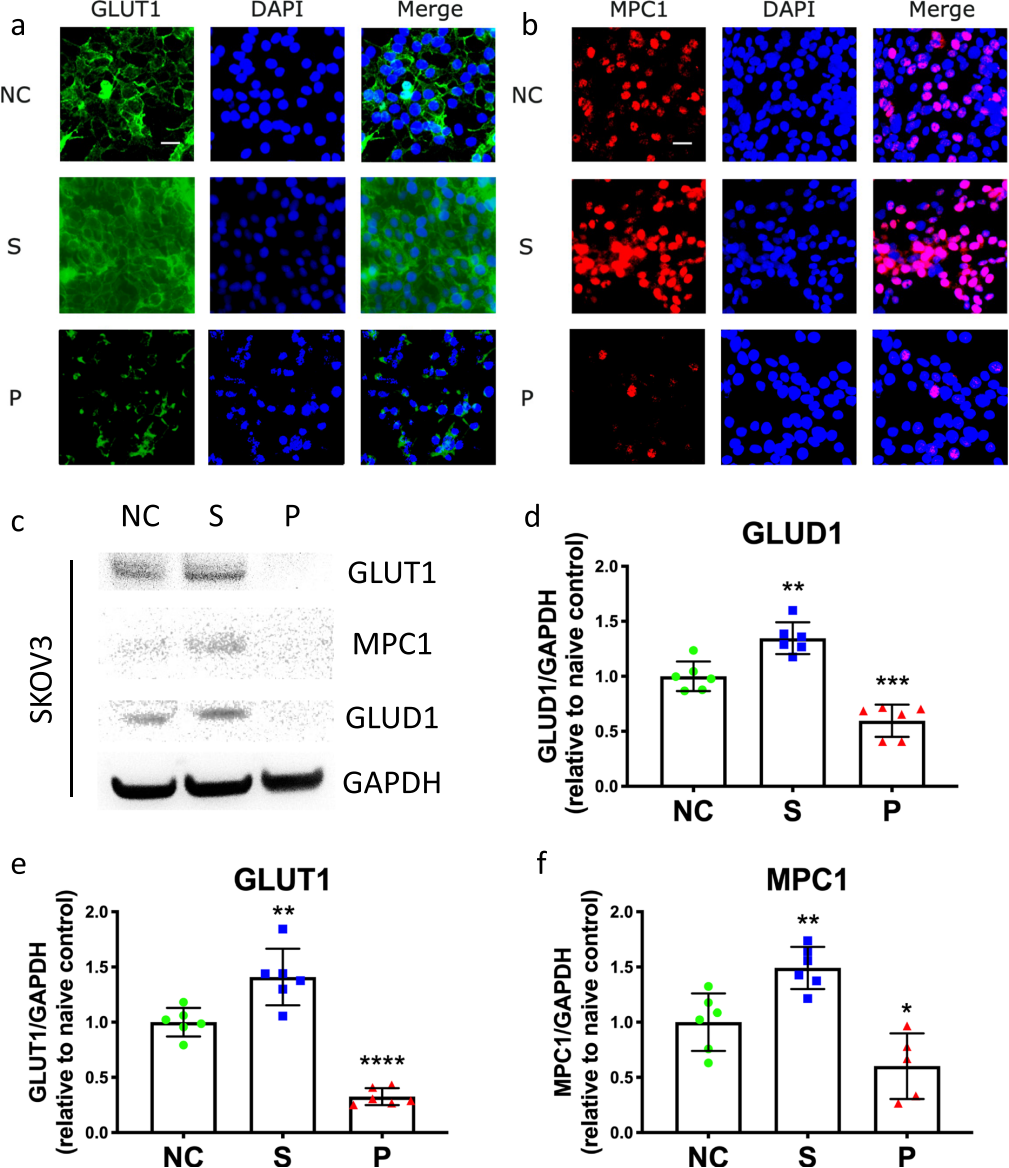
The effects of sevoflurane and propofol on GLUT1, MPC1, and GLUD1 in ovarian cancer cells. SKOV3 cells were exposed to media (naïve control), 2.5% sevoflurane, or 4 μg/mL propofol. The expressions of GLUT1 (**a**) and MPC1 (**b**) were detected with immunofluorescent staining. The expression levels of these two markers were validated with Western blot analysis, and the expression level of GLUD1 was also analyzed (**c**). The intensity of the bands in Western blot analysis was normalized by housekeeping protein GAPDH. The data were analyzed with one-way ANOVA and Dunnett multi-comparison test (**d**–**f**) and was presented as mean ± standard deviation and dots plot (*n* = 6). *p < 0.05, **p < 0.01, ***p < 0.001, ****p < 0.0001 versus naïve control. Scale bar: 50 μm. NC, naïve control group; S, sevoflurane group; P, propofol group; GLUT1, glucose transporter 1; MPC1, mitochondrial pyruvate carrier 1; GLUD1, glutamate dehydrogenase 1; ANOVA, analysis of variance

Based on the immunofluorescent staining of cells, the intensities of GLUT1 (Fig. 2a) and MPC1 (Fig. 2b) were higher in the sevoflurane group compared to naïve control, but much lower in the propofol group than the naïve control. From Western blot analysis, the protein expression levels of GLUT1 (NC vs. S, 1.0 ± 0.1 vs. 1.4 ± 0.3, *p* = 0.002, *n* = 6) and MPC1 (NC vs. S, 1.0 ± 0.3 vs. 1.5 ± 0.2, *p* = 0.008, *n* = 6) were significantly increased after sevoflurane exposure (Fig. 2c). However, the expression levels of GLUT1 (NC vs. P, 1.0 ± 0.1 vs. 0.3 ± 0.1, *p* < 0.0001, *n* = 6) and MPC1 (NC vs. P, 1.0 ± 0.3 vs. 0.6 ± 0.3, *p* = 0.036, *n* = 6) were significantly decreased after propofol treatment compared to the naïve control (Fig. 2e and f). Similarly, the expression level of GLUD1 was significantly increased by sevoflurane administration (NC vs. S, 1.0 ± 0.1 vs. 1.4 ± 0.1, *p* = 0.001, *n* = 6), but decreased by propofol administration (NC vs. P, 1.0 ± 0.1 vs. 0.6 ± 0.2, *p* < 0.001, *n* = 6) (Fig. 2d).

### Effects of sevoflurane or propofol on the cellular metabolism

**Fig. 3:**
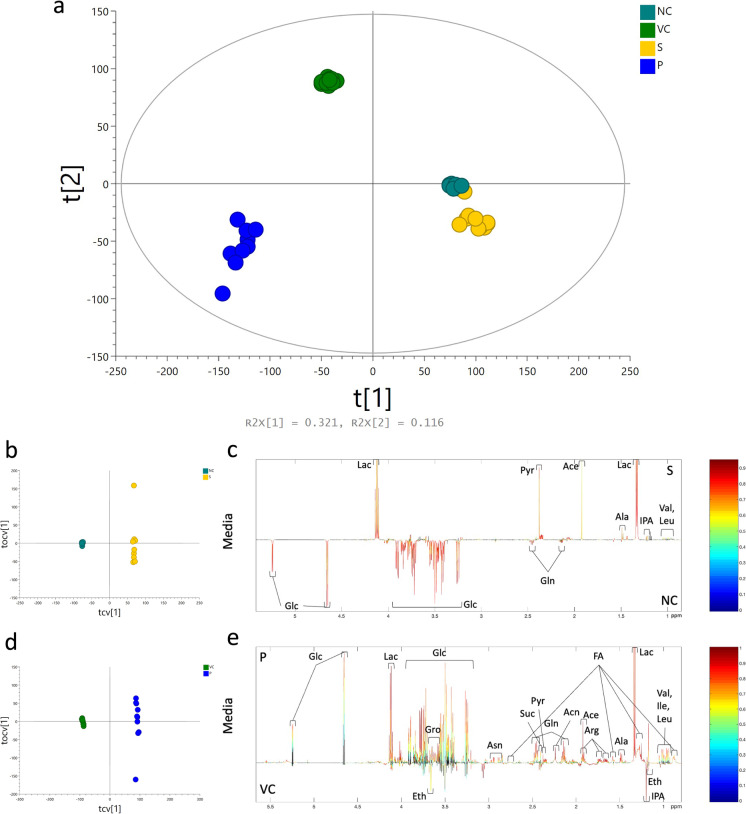
The metabolomics study of sevoflurane- or propofol-treated ovarian cancer cells using ^1^H-NMR spectroscopy. Ovarian cancer cells were administered with media (naïve control), intralipid (vehicle control), 2.5% sevoflurane or 4 μg/mL propofol for 2-h plus 24-h recovery time. After treatment, the culture media of ovarian cancer SKOV3 cells was collected for the ^1^H-NMR spectroscopy experiment. PCA scores plot of ^1^H-NMR spectra of media was shown (**a**). R^2^X represented the fraction of variance in the ^1^H-NMR data modeled by two principal components (t[1] and t[2]). The OPLS-DA scores plots (**b**: NC vs. S; **d**: VC vs. P) and loadings plots (**c**: NC vs. S; **e**: VC vs. P) were derived from ^1^H-NMR spectral data (*n* = 10). The color bar indicated the correlation coefficient values (*r*^2^) to be high in red and low in blue. NC, naïve control group; VC, vehicle control group; S, sevoflurane group; P, propofol group; PCA, principal component analysis; OPLS-DA, orthogonal projection to latent structures-discriminant analysis; Glc, glucose; Lac, lactate; Pyr, pyruvate; Gln, glutamine; Ace, acetate; Ala, alanine; IPA, isopropanol; Val, valine; Leu, leucine; Eth, ethanol; Gro, glycerol; Asn, asparagine; FA, fatty acids; Suc, succinate; Acn, acetone; Arg, arginine; Ile, isoleucine

The metabolic profiles obtained from media samples of naïve control, vehicle control, sevoflurane, and propofol groups were analyzed using unsupervised PCA analysis with two principal components. The PCA scores plot showed a clear grouping pattern among these four groups (Fig. 3a). Pair-wise comparisons of naïve control *vs*. sevoflurane and vehicle control vs. propofol groups were carried out using OPLS-DA analysis with one predictive component and one orthogonal component. Permutation *p* values showed both models of naïve control vs. sevoflurane (*p* = 0.005) and vehicle control vs. propofol (*p* = 0.005) had significances. The permutation p values together with R^2^X, Q^2^X, and Q^2^Y of the two models were summarized (Table 1). The OPLS-DA scores plots showed clear separations between naïve control and sevoflurane group (Fig. 3b) and vehicle control and propofol group (Fig. 3d). The OPLS-DA loading plots showed that glucose and glutamine concentrations were decreased, and the concentration of isopropanol was increased in the sevoflurane group compared to the naïve control group (Fig. 3c). However, the concentrations of glucose and glutamine were increased, and the concentration of isopropanol was decreased in the propofol group in contrast to the vehicle control group (Fig. 3e). After anesthetics administration, the concentrations of lactate, pyruvate, acetate, alanine, valine, and leucine were increased by both sevoflurane and propofol. Furthermore, propofol also increased glycerol, fatty acids, asparagine, succinate, acetone, arginine, and isoleucine but decreased ethanol (Table 2).

**Table 1:** Summary of the parameters of the OPLS-DA models

Model	Type	O-PLS-DA statistical parameters			
		R^2^X	R^2^Y	Q^2^Y	Permutation *P* value
NC vs. S	Media	20.49%	99.87%	0.86	0.005
VC vs. P	Media	31.78%	99.91%	0.98	0.005

The models are based on the media spectral data of SKOV3 cell line comparing naïve control and sevoflurane treatment groups or vehicle control and propofol groups. Permutation *p* values were derived from 100 permutes (*n* = 10)

**Abbreviations:** *NC*, naïve control group; *VC*, vehicle control group; *S*, sevoflurane group; *P*, propofol group; *OPLS-DA*, orthogonal projections to latent structures discriminant analysis

**Table 2:** Summary of the metabolites that were present in the media of SKOV3 cells

Metabolites	δ^1^H	Model	*r*	*p*	*q*
Acetate	1.92 (s)	NC vs. S	0.85	4.44E-06	5.04E-05
		VC vs. P	0.99	6.22E-19	5.04E-16
Acetone	2.24 (s)	VC vs. P	0.99	2.11E-18	1.12E-15
Alanine	1.48 (d); 3.78 (q)	NC vs. S	0.95	2.53E-10	1.45E-07
		VC vs. P	0.97	4.74E-12	1.59E-10
Arginine	1.68 (m), 1.90 (m), 3.23 (t), 3.76 (t)	VC vs. P	0.99	3.18E-15	4.05E-13
Asparagine	2.86 (dd), 2.96 (dd), 4.00 (dd)	VC vs. P	0.97	3.18E-12	1.13E-10
Ethanol	1.17 (t), 3.65 (q)	VC vs. P	− 0.99	5.70E-18	2.50E-15
Fatty acids	0.88 (m); 1.28 (m); 1.58 (m); 2.04 (m); 2.25 (m)	VC vs. P	0.88	3.25E-07	2.54E-06
Glucose	3.25 (t); 3.39–3.55 (m); 3.69–3.93 (m); 4.65 (d); 5.24 (d)	NC vs. S	− 0.9	1.18E-07	2.99E-06
		VC vs. P	0.91	3.66E-08	3.79E-07
Glutamine	2.14 (m); 2.44 (m); 3.77 (m)	NC vs. S	− 0.97	3.75E-12	3.16E-08
		VC vs. P	0.98	2.22E-14	1.94E-12
Glycerol	3.55 (m); 3.64 (m); 3.78 (m)	VC vs. P	0.93	1.79E-09	2.74E-08
Isoleucine	0.94 (t); 1.01 (d); 1.27 (m); 1.48 (m); 3.67 (m)	VC vs. P	0.91	3.94E-08	4.04E-07
Isopropanol	1.18 (dd); 4.03 (m)	NC vs. S	0.65	2.37E-03	1.24E-02
		VC vs. P	− 0.82	8.06E-06	4.34E-05
Lactate	1.33 (d); 4.11 (q)	NC vs. S	0.92	3.77E-08	1.58E-06
		VC vs. P	0.99	1.35E-15	2.04E-13
Leucine	0.96 (d); 0.97 (d); 1.69 (m); 1.71 (m); 3.74 (t)	NC vs. S	0.82	1.58E-05	1.49E-04
		VC vs. P	0.93	3.08E-09	4.36E-08
Pyruvate	2.38 (s)	NC vs. S	0.89	3.74E-07	6.72E-06
		VC vs. P	0.95	1.93E-10	3.85E-09
Succinate	2.41 (s)	VC vs. P	0.84	3.47E-06	2.04E-05
Valine	0.99 (d); 1.04 (d); 2.27 (m); 3.61 (d)	NC vs. S	0.82	2.06E-05	1.88E-04
		VC vs. P	0.91	9.80E-09	1.21E-07

For each model, “ + ” indicates a higher correlation in the treated groups, whereas “ − “ indicates a higher correlation in the control groups. The symbol *r* represents the correlation coefficient values; *p* represents significance level based on two-tailed heteroscedastic *t*-test; *q* is corrected values using Benjamini–Hochberg correction

**Abbreviations:** *s*, singlets; *d*, doublets; *dd*, double of doublets; *t*, triplets; *q*, quartets; *m*, multiplets

### Regulations of sevoflurane or propofol on PEDF expression, Erk pathway, and HIF-1α expression

**Fig. 4:**
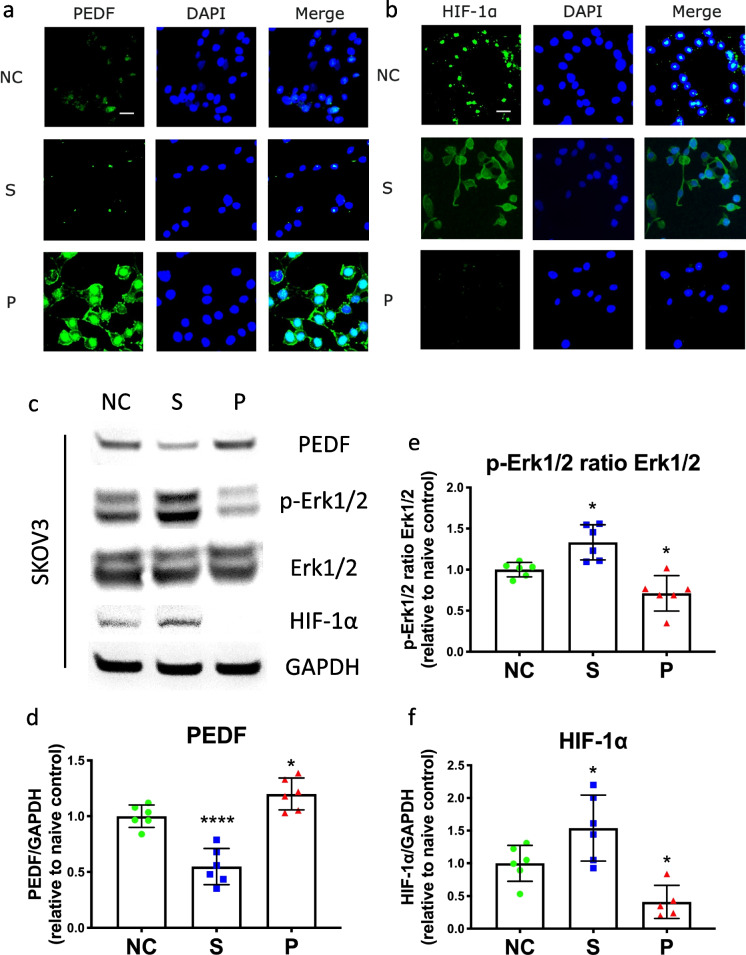
The opposite effects of sevoflurane and propofol on the PEDF/Erk/HIF-1α pathway in ovarian cancer cells. Ovarian cancer cells were administered with media (naïve control), 2.5% sevoflurane, or 4 μg/mL propofol. The expressions of PEDF (**a**) and HIF-1α (**b**) were detected with immunofluorescence. The expression levels of PEDF, p-Erk1/2, Erk1/2, and HIF-1α were evaluated with Western blot analysis (**c**). The intensity of PEDF and HIF-1α bands was normalized by GAPDH (**d** and **f**). At the same time, the intensity of the p-Erk1/2 band was normalized by Erk1/2 (**e**). Data were analyzed with one-way ANOVA with the Dunnett multi-comparison test and presented as mean ± standard deviation and dots plot (*n* = 6). *p < 0.05, ****p < 0.0001 versus naïve control. Scale bar: 50 μm. NC, naïve control group; S, sevoflurane group; P, propofol group; PEDF, pigment epithelium-derived factor; p-Erk1/2, phospho-extracellular-signal-regulated kinase 1 and 2; HIF-1α, hypoxia-inducible factor-1 alpha; ANOVA, analysis of variance

From immunofluorescent staining, it was found that the fluorescent intensity of PEDF was decreased in the sevoflurane group but increased in the propofol group compared to naïve control (Fig. 4a). The result was validated using Western blot analysis, which showed the significantly lower expression level of PEDF after sevoflurane exposure (NC vs. S, 1.0 ± 0.1 vs. 0.6 ± 0.2, *p* < 0.0001, *n* = 6) but significantly higher after propofol exposure (NC vs. P, 1.0 ± 0.1 vs. 1.2 ± 0.1, *p* = 0.043, *n* = 6) (Fig. 4d). Different from PEDF expression levels, the Western blot analysis showed that the expression level ratio of p-Erk1/2 to Erk1/2 was significantly increased in the sevoflurane group (NC vs. S, 1.0 ± 0.1 vs. 1.3 ± 0.2, *p* = 0.012, *n* = 6), but decreased in the propofol group compared to the control (NC vs. P, 1.0 ± 0.1 vs. 0.7 ± 0.2, *p* = 0.028, *n* = 6) (Fig. 4e). Similarly, the fluorescent staining showed SKOV3 cells administered with sevoflurane had a higher intensity of HIF-1α than the naïve control, while those of the propofol group had a lower intensity of HIF-1α than control (Fig. 4b). From Western blot analysis, it was also found that the expression level of HIF-1α was significantly increased after cancer cells administered with sevoflurane (NC vs. S, 1.0 ± 0.3 vs. 1.5 ± 0.5, *p* = 0.045, *n* = 6), but significantly decreased after propofol treatment compared to the control (NC vs. S, 1.0 ± 0.3 vs. 0.4 ± 0.3, *p* = 0.037, *n* = 6) (Fig. 4f).

### Effects of sevoflurane or propofol on CXCL12 and CXCR4 expressions

**Fig. 5:**
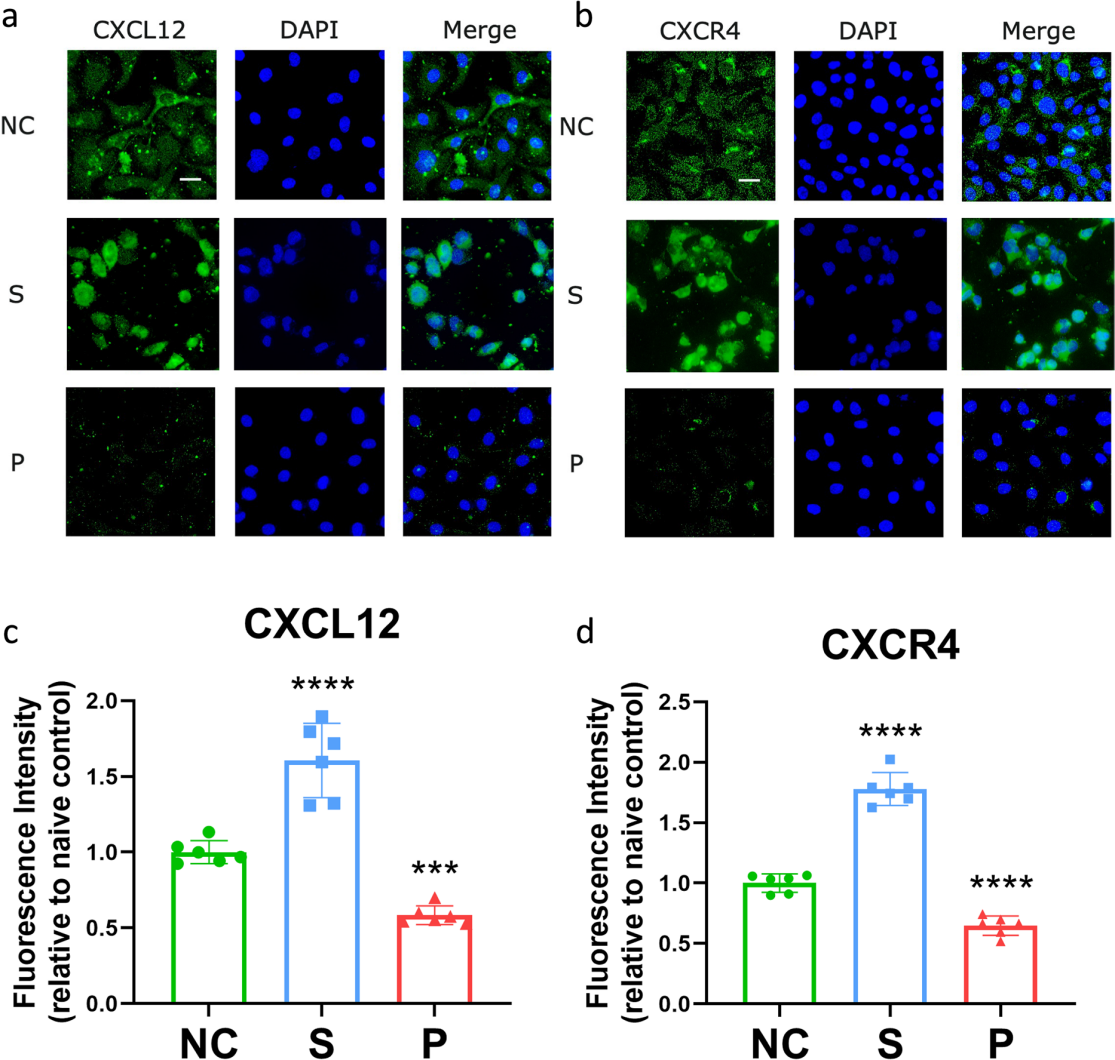
The expressions of CXCL12 and CXCR4 in ovarian cancer cells after sevoflurane or propofol exposure. SKOV3 cells were exposed to pure culture media (naïve control), 2.5% sevoflurane, or 4 μg/mL propofol for 2 h and followed by 24-h recovery. The expressions of CXCL12 (**a**) and CXCR4 (**b**) were detected with immunofluorescent staining (green) and overlaid with DAPI (blue). Sevoflurane exposure increased CXCL12 (**c**) and CXCR4 (**d**) in ovarian cancer cells, while propofol decreased the expressions of both markers. NC, naïve control group; S, sevoflurane group; P, propofol group; CXCL12, C-X-C motif chemokine ligand 12; CXCR4, C-X-C motif chemokine receptor 4

According to immunofluorescent staining, the intensity of CXCL12 was increased in the sevoflurane group but decreased in the propofol group (Fig. 5a and c). Similarly, the fluorescent staining of CXCR4 was higher in the sevoflurane group than the control, and the intensity of the marker was lower in the propofol group than the control (Fig. 5b and d). These results showed that the expressions of CXCL12 and CXCR4 were both increased by sevoflurane but decreased by propofol administration. The original data was included in Table S2.

## Discussion

**Fig. 6:**
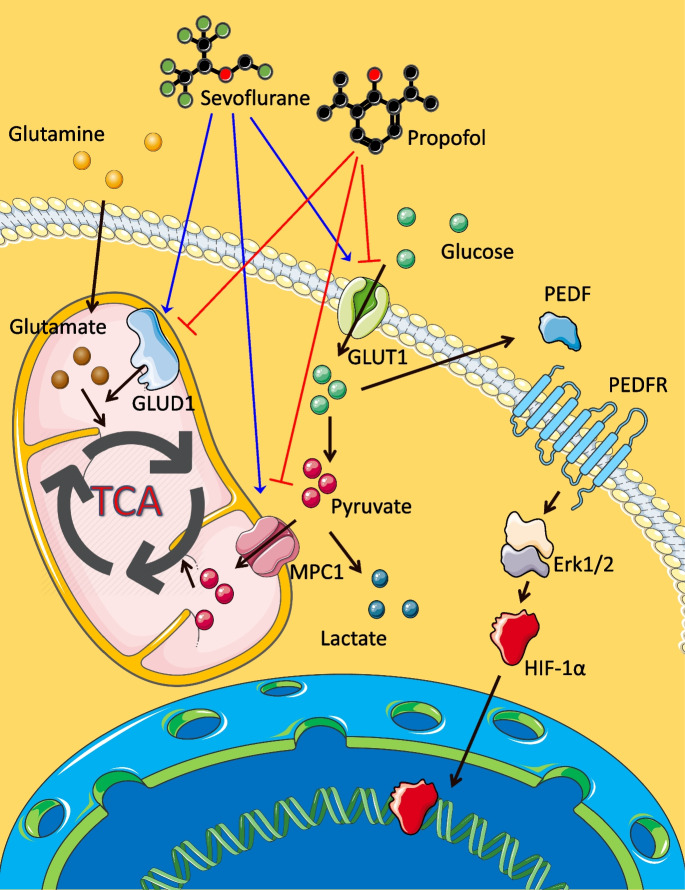
The molecular interactions in ovarian cancer cells after anesthetic administration. Sevoflurane upregulated the expressions of GLUT1, MPC1, and GLUD1, which induced glucose uptake, pyruvate mitochondrial transportation, and glutaminolysis. These processes enhanced the TCA cycle activity and decreased the PEDF expression. The downregulation of PEDF upregulated the HIF-1α via the Erk1/2 pathway. The role of HIF-1α was as a transcriptional regulator, which upregulated tumor-related genes. However, propofol had an opposite function on the above biomarkers. GLUT1, glucose transporter 1; MPC1, mitochondrial pyruvate carrier 1; GLUD1, glutamate dehydrogenase 1; PEDF, pigment epithelium-derived factor; p-Erk1/2, phospho-extracellular-signal-regulated kinase 1 and 2; HIF-1α, hypoxia-inducible factor-1 alpha

Our current study suggested that inhalational anesthetic sevoflurane enhanced ovarian cancer cell viability, proliferation, migration, and invasion. In contrast, intravenous anesthetic propofol inhibited those cellular activities. These phenotypic changes may be correlated with the upregulated expressions of GLUT1, MPC1, GLUD1, p-Erk1/2, HIF-1α, CXCL12 and CXCR4, and the downregulated PEDF expression by sevoflurane. Sevoflurane, but not propofol, promoted the energic metabolism efficiency of ovarian cancer cells by enhancing the uptake of the metabolic substrates such as glucose and glutamine and improved their growth and malignancy (Fig. 6).

Clinically, patients are administered with propofol for maintenance of general anesthesia under target plasma concentration of propofol of 4 μg/mL (Zheng et al. 2022), while 2–3% sevoflurane is usually given to patients for anesthesia maintenance (Yancik 1993; Nickalls and Mapleson 2003). In addition, most surgery lasts for 2–3 h. Therefore, our data reported here were achieved under that the dose or concentration and anesthetic exposure time are all clinically relevant.

The lower concentration of glucose and higher concentration of pyruvate in the media were found after sevoflurane exposure compared to the naïve control, which was in line with the upregulated expression level of GLUT1. The enhanced activity of GLUT1 may transport more glucose into the cytoplasm to convert to pyruvate, which explained the metabolic changes in the media after sevoflurane administration. In contrast, propofol downregulated GLUT1, which resulted in less glucose uptake from media, and hence, the concentration of glucose was increased after propofol exposure. The cellular glucose uptake via a high level of GLUT1 expression was correlated with the malignancy of cancers (Leung 2007; Pezzuto et al. 2020) and the overexpression of GLUT1 in cancer cells was essential for the high rate of glycolysis (Wright 2020). Propofol was reported to downregulate the *GLUT1* gene in macrophages (Tanaka et al. 2010) and this was due to the downregulation of GLUT1 proteins in the rat brain tissue under hypoxic preconditioning (Xiao et al. 2020). This condition mimics the cancer cell scenario: under high proliferative rate, cancer cells had inadequate oxygen supply and were under hypoxic conditions (Mudassar et al. 2020). These support our findings that the malignancy of ovarian cancer cells was related to the expression of GLUT1 after anesthetic administration.

The concentration of lactate was increased in both sevoflurane and propofol treatments, resulting from the increased glycolysis of pyruvate and thus subsequently more lactate being generated. It was reported that sevoflurane and propofol both increased the lactate level in the blood of dogs (Söbbeler et al. 2018). Another study in mice also demonstrated that sevoflurane increased pyruvate and lactate levels (Horn and Klein 2010). All these reports were in line with the findings of our current study.

It was found that both MPC1 and GLUD1 expressions were upregulated after sevoflurane exposure but were downregulated after propofol treatment. Besides, the concentration of glutamine in media was decreased after sevoflurane administration. MPC1, a member of the mitochondrial carrier system, locates at the inner membrane of mitochondria and transports pyruvate into mitochondria from the cytoplasm (Taylor 2017). The expression of MPC1 is decreased in most tumor types, especially those under a high proliferation rate related to the increased rate of glycolysis. This suggests that pyruvate likely shifts from the mitochondrial TCA cycle to cytoplasm glycolysis, which does not require oxygen supply as cancer cells are usually under hypoxic conditions (Rauckhorst and Taylor 2016). In addition, glutaminolysis compensates for the disturbed function of the TCA cycle due to less pyruvate intake and cancer cells, in turn, uptake glutamine and convert them into glutamate. The glutamate can be converted to α-ketoglutarate under the activation of GLUD1 and used by the TCA cycle to restore the survival of cancer cells as the intermediates of the TCA cycle are the source for the synthesis of amino acids, proteins, fatty acids, lipids, carbon skeleton, and nucleic acids (Yoo et al. 2020). Evidence from other studies showed propofol might disturb the mitochondrial respiratory chain, which was related to the TCA cycle (Berndt et al. 2018). It was also reported that unlike the inhibitory effects of propofol, sevoflurane preserved the mitochondrial respiratory chain function in a myocardial ischemic model (Lotz et al. 2020). Our data demonstrated that after sevoflurane administration, the MPC1 and GLUD1 expressions were upregulated, enhancing the activity of the TCA cycle to meet the demands of cancer survival and progression. The decreased glutamine concentration in media suggested that the utilization of glutamine was likely increased, and glutaminolysis was then promoted. With the disturbed function of the TCA cycle after propofol exposure, amino acids that can be used in the TCA cycle were accumulated, such as asparagine and arginine (Pasini et al. 2018), which was consistent with our findings.

Another “mirror change” of metabolites between sevoflurane and propofol administration was isopropanol except for glucose and glutamine. It was reported that the level of isopropanol was increased in the exhaled breath of lung cancer patients, and it had been regarded as a potential biomarker for lung cancer diagnosis (Chien et al. 2017). It seemed the level of isopropanol had some correlations with cancer malignancy, which was consistent with the findings of this study that sevoflurane enhanced the malignancy of ovarian cancer cells and increased the level of isopropanol, while propofol inhibited the malignancy of ovarian cancer cells and decreased the level of isopropanol. Isopropanol can be reversibly converted to acetone (Beauchamp et al. 2016; Li et al. 2017), which may also contribute to the increased level of acetone in the media of the propofol group.

The levels of glycerol and fatty acids were increased in the propofol group. Through *β*-oxidation of fatty acids, acetyl-CoA is generated and used in the TCA cycle (Liu 2006). Thus, the changes of glycerol and fatty acids in the propofol group were another evidence that the mitochondria function and TCA cycle was inhibited or disturbed by propofol treatment. The glycerol and fatty acids might also come from cell membrane degradation and phospholipid metabolism. From an earlier study, it was found that propofol affected the membrane ultrastructure of HeLa cells that the surface roughness of cellular membrane was decreased in a dose-dependent manner (Zhang et al. 2016).

In the current study, the expression level of PEDF was decreased after sevoflurane administration but increased after propofol treatment. In human retinal pigment epithelium, the expression of GLUT1 was increased under hypoxia conditions that resulted in the increased uptake of glucose, which led to a decrease of PEDF expression (Calado et al. 2016a, b). Another study also reported that the *PEDF* gene overexpression in mice was related to reducing glucose uptake and decreased expression of GLUT1 (Calado et al. 2016a, b). These reports were consistent with our findings that sevoflurane increased the GLUT1 expression and glucose uptake, which led to a downregulated expression of PEDF. However, an opposite effect was found with propofol treatment.

In the current study, the Erk1/2 signaling pathway was induced after sevoflurane exposure but inhibited after propofol exposure. There was evidence that the increased PEDF expression was related to the Erk1/2 signaling pathway inhibition in a diabetic model (Dong et al. 2019), which was in line with our results. HIF-1α is a transcriptional factor regulated by various signaling pathways, including the Erk1/2 pathway (Liu et al. 2020). In cancer cells, the HIF-1α is overexpressed, which regulates tumor survival-related genes, such as *CXCL12* and *CXCR4* (Gola et al. 2020; Xue et al. 2020). It was in line with the current study data that sevoflurane upregulated the Erk1/2 signaling pathway and HIF-1α, CXCL12, and CXCR4 expressions while propofol downregulated these molecular entities.

Our study has some limitations. Firstly, the cause and effect between cellular signaling changes and metabolic alterations induced by anesthetics remains unknown. However, it is very likely that, for example, sevoflurane promotes cancer cell survival and development due to cell survival signaling pathway activation and, subsequently, metabolic changes occurred. Secondly, how our cultured cell study related to human disease is not known. However, retrospective clinical studies indicated that breast, colonic, and rectal cancer patients were anesthetized with inhalational anesthetics sevoflurane or desflurane, or intravenous anesthetic propofol during surgery, and the survival rate of propofol anesthetized patients were significantly higher than those with inhalational anesthesia (Enlund et al. 2014; Wu et al. 2018). Laboratory data, including the one reported here, and retrospective clinical data, all point to that sevoflurane might be a risk factor for cancer patients, while propofol may benefit cancer patients for their surgery. Therefore, clinical studies are urgently needed to optimize anesthesia regimens for better surgical outcomes for cancer patients. Thirdly, it might be worth assessing a longer recovery period and at different time points after anesthetic exposure with colony survival assay. However, our study was designed to mimic clinical settings for surgery with relative short exposure and recovery period of anesthetics use. The conclusion derived from our data reported here is likely valid clinically per se. Fourthly, other cancer cell lines and/or normal cell lines should be used and other biomarkers, for example, nuclear factor erythroid 2-related factor 2 (NRF2) and p53, should be determined in the present study as well. Propofol inhibited the cell viability, proliferation, migration, and invasion of lung cancer cells; all of which were associated with several pro-tumor genes (such as VEGFA, CTBP1 and CST7) were downregulated but several anti-tumor genes (such as NR4A3, RB1, and NME1) of lung cancer cells were upregulated after propofol treatment. However, these were not noticed on neuroglioma cells (Hu et al. 2021). These indicate that the cancer cell biology of anesthetics depends on different cancer cells, and thus, further study is needed.

## Conclusions

The current study demonstrated that sevoflurane upregulated GLUT1, MPC1, and GLUD1 expressions of ovarian cancer cells, while propofol downregulated the expressions of these molecules. These regulations by sevoflurane or propofol on ovarian cancer cells led to different metabolic features. The upregulated GLUT1 expression and, in turn, increased glucose uptake after sevoflurane exposure resulted in a decreased expression of PEDF. In contrast, an increased expression of PEDF was found after propofol treatment. Furthermore, unlike propofol, sevoflurane upregulated the Erk1/2 pathway, HIF-1α, CXCL12, and CXCR4 expressions through PEDF inhibition per se. In summary, the profiling alterations of molecular and metabolic modulations found in the present study indicate the pro- and anti-tumor properties of sevoflurane and propofol, respectively. The translational value of these findings should be further studied in pre-clinical and clinical settings.

## Supplementary Information

Below is the link to the electronic supplementary material.

Supplementary file1 (DOCX 18 KB)

## Data Availability

The datasets generated during and/or analyzed during the current study are available from the corresponding author on reasonable request.

## References

[CR1] Beauchamp GA, Valento M, Kim J. Toxic alcohol ingestion: prompt recognition and management in the emergency department [digest]. Emerg Med Pract. 2016;18(9 Suppl Points & Pearls):S1-s2. 28745842

[CR2] Berndt N, Rösner J, Haq RU, Kann O, Kovács R, Holzhütter HG, Spies C, Liotta A (2018). Possible neurotoxicity of the anesthetic propofol: evidence for the inhibition of complex II of the respiratory chain in area CA3 of rat hippocampal slices. Arch Toxicol.

[CR3] Calado SM, Alves LS, Simão S, Silva GA (2016). GLUT1 activity contributes to the impairment of PEDF secretion by the RPE. Mol vis.

[CR4] Calado SM, Diaz-Corrales F, Silva GA (2016). pEPito-driven PEDF expression ameliorates diabetic retinopathy hallmarks. Hum Gene Ther Methods.

[CR5] Chien PJ, Suzuki T, Tsujii M, Ye M, Toma K, Arakawa T, Iwasaki Y, Mitsubayashi K (2017). Bio-sniffer (gas-phase biosensor) with secondary alcohol dehydrogenase (S-ADH) for determination of isopropanol in exhaled air as a potential volatile biomarker. Biosens Bioelectron.

[CR6] Craze ML, El-Ansari R, Aleskandarany MA, Cheng KW, Alfarsi L, Masisi B, Diez-Rodriguez M, Nolan CC, Ellis IO, Rakha EA, Green AR (2019). Glutamate dehydrogenase (GLUD1) expression in breast cancer. Breast Cancer Res Treat.

[CR7] Dieterle F, Ross A, Schlotterbeck G, Senn H (2006). Probabilistic quotient normalization as robust method to account for dilution of complex biological mixtures. Application in 1H NMR metabonomics. Anal Chem.

[CR8] Dong Y, Wan G, Yan P, Qian C, Li F, Peng G (2019). Fabrication of resveratrol coated gold nanoparticles and investigation of their effect on diabetic retinopathy in streptozotocin induced diabetic rats. J Photochem Photobiol B.

[CR9] Enlund M, Berglund A, Andreasson K, Cicek C, Enlund A, Bergkvist L (2014). The choice of anaesthetic–sevoflurane or propofol–and outcome from cancer surgery: a retrospective analysis. Ups J Med Sci.

[CR10] Ferlay J, Ervik M, Lam F, Colombet M, Mery L, Piñeros M, Znaor A, Soerjomataram I, Bray F. Global cancer observatory: cancer today. Lyon, France: International Agency for Research on Cancer. 2020;Retrieved June 4th, 2022, from https://gco.iarc.fr/today.

[CR11] Filleur S, Nelius T, de Riese W, Kennedy RC (2009). Characterization of PEDF: a multi-functional serpin family protein. J Cell Biochem.

[CR12] Gola C, Iussich S, Noury S, Martano M, Gattino F, Morello E, Martignani E, Maniscalco L, Accornero P, Buracco P, Aresu L, De Maria R (2020). Clinical significance and in vitro cellular regulation of hypoxia mimicry on HIF-1α and downstream genes in canine appendicular osteosarcoma. Vet J.

[CR13] Haggart GA. csmsoftware/IMPaCTS: Version 1.1.1 (Version v1.1.1). 2019;from 10.5281/zenodo.3077413.

[CR14] Horn T, Klein J (2010). Lactate levels in the brain are elevated upon exposure to volatile anesthetics: a microdialysis study. Neurochem Int.

[CR15] Horowitz M, Neeman E, Sharon E, Ben-Eliyahu S (2015). Exploiting the critical perioperative period to improve long-term cancer outcomes. Nat Rev Clin Oncol.

[CR16] Hu C, Iwasaki M, Liu Z, Wang B, Li X, Lin H, Li J, Li JV, Lian Q, Ma D (2021). Lung but not brain cancer cell malignancy inhibited by commonly used anesthetic propofol during surgery: Implication of reducing cancer recurrence risk. J Adv Res.

[CR17] Huang H, Benzonana LL, Zhao H, Watts HR, Perry NJ, Bevan C, Brown R, Ma D (2014). Prostate cancer cell malignancy via modulation of HIF-1α pathway with isoflurane and propofol alone and in combination. Br J Cancer.

[CR18] Iwasaki M, Zhao H, Jaffer T, Unwith S, Benzonana L, Lian Q, Sakamoto A, Ma D (2016). Volatile anaesthetics enhance the metastasis related cellular signalling including CXCR2 of ovarian cancer cells. Oncotarget.

[CR19] Karagiota A, Kourti M, Simos G, Mylonis I (2019). HIF-1α-derived cell-penetrating peptides inhibit ERK-dependent activation of HIF-1 and trigger apoptosis of cancer cells under hypoxia. Cell Mol Life Sci.

[CR20] Leung K. 8-[123I]Iodo-L-1,2,3,4-tetrahydro-7-hydroxyisoquinoline-3-carboxylic acid. In: Molecular Imaging and Contrast Agent Database (MICAD) [Internet]. National Center for Biotechnology Information. 2007. 20641893

[CR21] Li WW, Liu Y, Liu Y, Cheng SQ, Duan YX (2017). Exhaled isopropanol: new potential biomarker in diabetic breathomics and its metabolic correlations with acetone. RSC Adv.

[CR22] Liu Y (2006). Fatty acid oxidation is a dominant bioenergetic pathway in prostate cancer. Prostate Cancer Prostatic Dis.

[CR23] Liu RM, Xu P, Chen Q, Feng SL, Xie Y (2020). A multiple-targets alkaloid nuciferine overcomes paclitaxel-induced drug resistance in vitro and in vivo. Phytomedicine.

[CR24] Lotz C, Stumpner J, Smul TM (2020). Sevoflurane as opposed to propofol anesthesia preserves mitochondrial function and alleviates myocardial ischemia/reperfusion injury. Biomed Pharmacother.

[CR25] Mudassar F, Shen H, O’Neill G, Hau E (2020). Targeting tumor hypoxia and mitochondrial metabolism with anti-parasitic drugs to improve radiation response in high-grade gliomas. J Exp Clin Cancer Res.

[CR26] Nickalls RW, Mapleson WW (2003). Age-related iso-MAC charts for isoflurane, sevoflurane and desflurane in man. Br J Anaesth.

[CR27] Pasini E, Corsetti G, Aquilani R, Romano C, Picca A, Calvani R, Dioguardi FS. Protein-amino acid metabolism disarrangements: the hidden enemy of chronic age-related conditions. Nutrients. 2018;10(4):391. 10.3390/nu10040391 PMC5946176 29565819

[CR28] Pezzuto A, D'Ascanio M, Ricci A, Pagliuca A, Carico E. Expression and role of p16 and GLUT1 in malignant diseases and lung cancer: a review. Thorac Cancer. 2020;11(11):3060–3070. 10.1111/1759-7714.13651 PMC7606016 32945604

[CR29] Rauckhorst AJ, Taylor EB (2016). Mitochondrial pyruvate carrier function and cancer metabolism. Curr Opin Genet Dev.

[CR30] Saphner T, Tormey DC, Gray R (1996). Annual hazard rates of recurrence for breast cancer after primary therapy. J Clin Oncol.

[CR31] Scala S, D'Alterio C, Milanesi S, Castagna A, Carriero R, Farina FM, Locati M, Borroni EM. New insights on the emerging genomic landscape of CXCR4 in cancer: a lesson from WHIM. Vaccines (Basel). 2020;8(2):164. 10.3390/vaccines8020164 PMC7349554 32260318

[CR32] Söbbeler FJ, Carrera I, Pasloske K, Ranasinghe MG, Kircher P, Kästner SBR (2018). Effects of isoflurane, sevoflurane, propofol and alfaxalone on brain metabolism in dogs assessed by proton magnetic resonance spectroscopy ((1)H MRS). BMC Vet Res.

[CR33] Son J, Lyssiotis CA, Ying H, Wang X, Hua S, Ligorio M, Perera RM, Ferrone CR, Mullarky E, Shyh-Chang N, Kang Y, Fleming JB, Bardeesy N, Asara JM, Haigis MC, DePinho RA, Cantley LC, Kimmelman AC (2013). Glutamine supports pancreatic cancer growth through a KRAS-regulated metabolic pathway. Nature.

[CR34] Takano T, Li YJ, Kukita A, Yamaza T, Ayukawa Y, Moriyama K, Uehara N, Nomiyama H, Koyano K, Kukita T (2014). Mesenchymal stem cells markedly suppress inflammatory bone destruction in rats with adjuvant-induced arthritis. Lab Invest.

[CR35] Tanaka T, Takabuchi S, Nishi K, Oda S, Wakamatsu T, Daijo H, Fukuda K, Hirota K (2010). The intravenous anesthetic propofol inhibits lipopolysaccharide-induced hypoxia-inducible factor 1 activation and suppresses the glucose metabolism in macrophages. J Anesth.

[CR36] Taylor EB (2017). Functional properties of the mitochondrial carrier system. Trends Cell Biol.

[CR37] Trygg J, Holmes E, Lundstedt T (2007). Chemometrics in metabonomics. J Proteome Res.

[CR38] Veselkov KA, Lindon JC, Ebbels TM, Crockford D, Volynkin VV, Holmes E, Davies DB, Nicholson JK (2009). Recursive segment-wise peak alignment of biological 1H NMR spectra for improved metabolic biomarker recovery. Anal Chem.

[CR39] Wright EM (2020). SGLT2 and cancer. Pflugers Arch.

[CR40] Wu ZF, Lee MS, Wong CS, Lu CH, Huang YS, Lin KT, Lou YS, Lin C, Chang YC, Lai HC (2018). Propofol-based total intravenous anesthesia is associated with better survival than desflurane anesthesia in colon cancer surgery. Anesthesiology.

[CR41] Wyld L, Audisio RA, Poston GJ (2015). The evolution of cancer surgery and future perspectives. Nat Rev Clin Oncol.

[CR42] Xiao F, Lv J, Liang YB, Chen YH, Tu YB, Guan RC, Li L, Xie YB (2020). The expression of glucose transporters and mitochondrial division and fusion proteins in rats exposed to hypoxic preconditioning to attenuate propofol neurotoxicity. Int J Neurosci.

[CR43] Xue Y, Li Z, Wang Y, Zhu X, Hu R, Xu W (2020). Role of the HIF-1α/SDF-1/CXCR4 signaling axis in accelerated fracture healing after craniocerebral injury. Mol Med Rep.

[CR44] Yancik R (1993). Ovarian cancer. Age contrasts in incidence, histology, disease stage at diagnosis, and mortality. Cancer.

[CR45] Yoo HC, Yu YC, Sung Y, Han JM. Glutamine reliance in cell metabolism. Exp Mol Med. 2020;52(9):1496–1516. 10.1038/s12276-020-00504-8 PMC8080614 32943735

[CR46] Zhang F, Wang C, Cui Y, Li S, Yao Y, Ci Y, Wang J, Hou W, Wu A, Li E (2016). Effects of propofol on several membrane characteristics of cervical cancer cell lines. Cell Physiol Biochem.

[CR47] Zhao H, Iwasaki M, Yang J, Savage S, Ma D (2014). Hypoxia-inducible factor-1: a possible link between inhalational anesthetics and tumor progression?. Acta Anaesthesiol Taiwan.

[CR48] Zheng Z, Xue F, Wang H, He Y, Zhang L, Ma W, Zhang C, Guan Y, Ye F, Wen Y, Li X, Huang M, Huang W, Wang Z, Li J (2022). A single nucleotide polymorphism-based formula to predict the risk of propofol TCI concentration being over 4 μg mL(-1) at the time of loss of consciousness. Pharmacogenomics J.

[CR49] Zhu Z-J, Hu Y, Zhao Y-F, Chen X-Z, Chen L-Q, Chen Y-T (2011). Early recurrence and death after esophagectomy in patients with esophageal squamous cell carcinoma. Ann Thorac Surg.

[CR50] Zou H, Chen Q, Zhang A, Wang S, Wu H, Yuan Y, Wang S, Yu J, Luo M, Wen X, Cui W, Fu W, Yu R, Chen L, Zhang M, Lan H, Zhang X, Xie Q, Jin G, Xu C (2019). MPC1 deficiency accelerates lung adenocarcinoma progression through the STAT3 pathway. Cell Death Dis.

