# Supplementary material for: Sevoflurane but not propofol enhances ovarian cancer cell biology through regulating cellular metabolic and signaling mechanisms
Source: Cell Biol Toxicol. 2022 Oct 8;39(4):1395–411. doi: 10.1007/s10565-022-09766-6 (PMC10425485; doi:10.1007/s10565-022-09766-6)
Supplement: Supplementary file 1 — Supplementary file1 (DOCX 18 KB) [file 10565_2022_9766_MOESM1_ESM.docx]

**Table S1** Primary and secondary antibodies for immunofluorescent staining and Western blot

| **Name** | **No.** | **Concentration** | **Source** | **Company** |
| --- | --- | --- | --- | --- |
| Anti-GLUT1 antibody | ab40084 | IF, 1:1000;  WB, 1:2500 | Mouse monoclonal | Abcam, Cambridge, UK |
| Anti-MPC1 antibody | NBP1-91706 | IF, 1:200;  WB, 1:250 | Rabbit polyclonal | Novus Biologicals, Abingdon, UK |
| Anti-GLUD1 antibody | ab166618 | WB, 1:1000 | Rabbit monoclonal | Abcam, Cambridge, UK |
| Anti-PEDF antibody | ab180711 | IF, 1:200;  WB, 1:500 | Rabbit polyclonal | Abcam, Cambridge, UK |
| Anti-p-Erk1/2 antibody | 4370 | IF, 1:200;  WB, 1:2000 | Rabbit monoclonal | Cell signalling Technology, London, UK |
| Anti-Erk1/2 antibody | 9102 | IF, 1:200;  WB, 1:1000 | Rabbit polyclonal | Cell signalling Technology, London, UK |
| Anti-HIF-1α antibody | NB100-105 | IF, 1:200;  WB, 1:500 | Rabbit polyclonal | Novus Biologicals, Abingdon, UK |
| Anti-CXCL12 antibody | ab155090 | IF, 1:250 | Rabbit monoclonal | Abcam, Cambridge, UK |
| Anti-CXCR4 antibody | ab124824 | IF, 1:500 | Rabbit monoclonal | Abcam, Cambridge, UK |
| Anti-GAPDH antibody | MAB374 | WB, 1:20000 | Mouse monoclonal | Merck, Hertfordshire, UK |
| Anti-Ki-67 antibody | sc-15402 | IF, 1:200 | Rabbit polyclonal | Santa Cruz Biotechnology, Dallas, Texas, USA |
| Anti-rabbit IgG H&L antibody | ab97075 | IF, 1:1000 | Goat polyclonal | Abcam, Cambridge, UK |
| Anti-mouse IgG H&L antibody | ab97035 | IF, 1:1000 | Goat polyclonal | Abcam, Cambridge, UK |
| Anti-rabbit IgG, HRP-linked antibody | 7074 | WB, 1:1000 | Horse | Cell signalling Technology, London, UK |
| Anti-mouse IgG, HRP-linked antibody | 7076 | WB, 1:1000 | Horse | Cell signalling Technology, London, UK |

**Abbreviations:** IF, immunofluorescence; WB, Western blotting.

**Table S2** Original data of CXCL12 and CXCR4 immunofluorescent staining

| **CXCL12** | **NC** | **S** | **P** |
| --- | --- | --- | --- |
| **1** | 50.163 | 77.355 | 26.885 |
| **2** | 48.433 | 64.114 | 25.642 |
| **3** | 45.691 | 83.271 | 29.058 |
| **4** | 54.937 | 87.091 | 33.869 |
| **5** | 46.901 | 63.457 | 27.927 |
| **6** | 44.78 | 91.914 | 26.372 |

| **CXCR4** | **NC** | **S** | **P** |
| --- | --- | --- | --- |
| **1** | 62.235 | 105.11 | 35.848 |
| **2** | 54.904 | 108.095 | 40.151 |
| **3** | 63.77 | 98.043 | 42.228 |
| **4** | 54.194 | 108.073 | 40.213 |
| **5** | 62.684 | 122.133 | 44.784 |
| **6** | 64.178 | 102.487 | 31.284 |
